# Comprehensive Flavor Analysis of Volatile Components During the Vase Period of Cut Lily (*Lilium* spp. ‘Manissa’) Flowers by HS-SPME/GC–MS Combined With E-Nose Technology

**DOI:** 10.3389/fpls.2022.822956

**Published:** 2022-06-17

**Authors:** Lijuan Wei, Shouhui Wei, Dongliang Hu, Li Feng, Yayu Liu, Huwei Liu, Weibiao Liao

**Affiliations:** College of Horticulture, Gansu Agricultural University, Lanzhou, China

**Keywords:** cut lily flowers, postharvest, volatile components, E-nose, HS-SPME/GC-MS, OAVs

## Abstract

Volatile compounds could affect the flavor and ornamental quality of cut flowers, but the flavor change occurring during the vase period of the cut flower is unclear. To clarify the dynamic changes during the vase period of cut lily (*Lilium* spp. ‘Manissa’) flowers, comprehensive flavor profiles were characterized by the electronic nose (E-nose) and headspace solid-phase microextraction gas chromatography-mass spectrometry (HS-SPME/GC-MS). The response value of sensor W2W was significantly higher than other sensors, and its response value reached the highest on day 4. A total of 59 volatiles were detected in cut lilies by HS-SPME/GC-MS, mainly including aldehydes, alcohols, and esters. There were 19 volatiles with odor activity values (OAVs) greater than 1. Floral and fruity aromas were stronger, followed by a pungent scent. Principal component analysis (PCA) and hierarchical cluster analysis (HCA) could effectively discriminate lily samples derived from different vase times on the basis of E-nose and HS-SPME-GC-MS. In summary, our study investigates the flavor change profile and the diversity of volatile compounds during the vase period of cut lilies, and lilies on day 4 after harvest exhibited excellent aroma and flavor taking into consideration of the flavor intensity and diversity. This provided theoretical guidance for the assessment of scent volatiles and flavor quality during the vase period of cut lily flowers and will be helpful for the application of cut lilies during the postharvest process.

## Introduction

Cut lilies (*Lilium* spp.) are bulbous plants with large, trumpet-shaped, and typically fragrant flowers and derived from interspecific crosses of species belonging to the Archelirion section (Liao et al., 2013). Additionally, lilies are well-known ornamental plants with a diversity of fragrant types (Du et al., 2019). Nowadays, cut lilies are in demand worldwide due to their superior commercial and ornamental value (Zhang et al., 2018a). The Netherlands dominated the world’s total trade in lily bulbs, with a production area of 5,280 hectares in 2020 (Kang et al., 2021). More than 600 cultivars of lily bulbs were produced in the Netherlands every year and exported to other countries (Asjes, 2000; Wani et al., 2016). In addition to the ornamental value, lily flowers have also extensive edible and medicinal value (Francis et al., 2004; Mlcek and Rop, 2011). Lily flowers have been cultivated for the production of lily oil and lily water for the treatment of skin and articular diseases (Pieroni, 2000; Zaccai et al., 2020). In recent years, the essential oil extracted from lily flowers has become more popular, which can effectively eliminate body odor and promote metabolism and detoxification. Since lily essential oil contains natural plant protein and polysaccharides, it also has an effect on moisturizing, soothing, and skin elasticity, accelerating cell growth and blood circulation (Bruni et al., 1997; Zaccai et al., 2020).

The postharvest vase process of cut lily flowers involves a complex process of maturation and senescence. In general, cut lily flowers are harvested at the tight and green bud (puffy bud) stage, which is also called as commercial harvest stage of cut lily flowers (Zhang et al., 2018a). From a physiological perspective, the development of cut lily flowers is a metabolic process, which involves the macromolecules, including protein, hormone, starch, lipid, and related inorganic ions (Zhang et al., 2021). Previously, various studies have also indicated the key regulatory mechanism of developmental processes in cut flowers. The flower opening, senescence, dynamics in various physiological and biochemical levels, and changes in organelle and cell levels have been elucidated (Ma et al., 2018; Zhang et al., 2018b). Interestingly, various previous results in our group also indicated that key signal molecules, such as hydrogen gas (Ren et al., 2017), nitric oxide, calcium, and hydrogen peroxide (Zhang et al., 2018a), not only regulated the metabolic process of cut lily after postharvest but also improved the vase life and ornamental quality.

Recently, electronic nose (E-nose) technology and headspace solid-phase microextraction combined with gas chromatography-mass spectrometry (HS-SPME/GC-MS) are applied in the food industry for quality control. E-nose is sensitive to smell information from samples, and slight changes in volatile compounds could cause the different responses of sensors (Emre and Bekir, 2017). Moreover, E-nose is designed as an apparatus to mimic the human olfactory perception within an instrument designed to obtain repeatable measurements, allowing identifications and classifications of aroma mixtures while eliminating the common occurrence of olfactory fatigue in people (Wilson and Ba Ietto, 2011). For example, E-nose analysis was generally supplied to comprehensively evaluate the overall information scent, thereby achieving fresh grading and quality control of vegetables, fruits, beverages, cereals, and meats (Wang et al., 2017; Mirzaee-Ghaleh et al., 2019). The HS-SPME/GC-MS technology has been widely applied in the identification of volatile compounds, which was characterized by easy control, good repeatability, low threshold, solvent-free, and sensitive approach (Wei et al., 2021).

*Lilium*, a famous and significant cut flower, emits a variety of volatile organic compounds (Zhang et al., 2018b). The volatile substances in different tissues of lily might be different. Johnson et al. (2016) indicated that floral volatile emission of *Lilium* was differential between tepals and reproductive organs, and tepal tissues (two petal whirls) emitted the vast majority of total volatile molecules compared with the reproductive organs of the flower. Moreover, for *Murraya koenigii* L., the stems and flowers have a strong aroma compared with the leaves (Walde et al., 2006). In addition, Shi et al. (2018) captured the changes in flower volatile emissions at four flower developmental stages of *Lilium* ‘Siberia’ and found that most of the flower scent compounds had the highest emission rate at the full-flowering stage. The accumulation levels of 80 out of 100 volatiles of *Rosa damascena* were significantly influenced by the flower developmental stages (Rusanov et al., 2011). These results reveal that the biosynthesis of floral volatiles was controlled developmentally in flowers. However, no literature is available on the assessment and change of scent volatiles and flavor quality during the vase period of cut flowers to date.

Thus, the objectives of this study were as follows: (1) to explore the overall flavor profile by E-nose during the vase period of lilies; (2) to identify and quantify the volatile compounds of cut lilies derived from different vase times by HS-SPME/GC–MS technology; (3) to further seek the characteristic odor compositions (OAVs greater than 1) in cut lilies and determine the main aroma types; (4) to distinguish the cut lilies derived from different vase times through radar fingerprint chart, hierarchical cluster analysis (HCA), and principal component analysis (PCA) on the basis of E-nose and HS-SPME-GC-MS; and (5) to evaluate and compare the odor compounds, aromatic profile and aroma, and flavor of lily flowers during the vase period. These results made a significant contribution to assess scent volatiles and ornamental quality during the vase period of cut lily flowers. By extension, it also powerfully provided a theoretical foundation for the development and application of cut lilies based on the volatile components of the commercial harvest stage.

## Materials and Methods

### Plant Materials and Experimental Conditions

**FIGURE 1:**
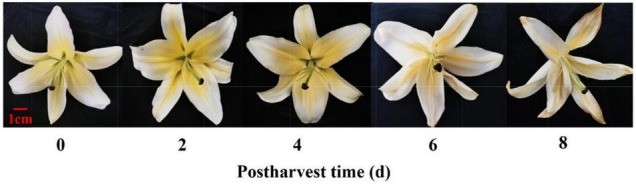
Physical phenotype of cut lily flowers after postharvest. Photographs were taken after postharvest 0, 2, 4, 6, and 8 days, respectively.

The experiment was conducted using lilies (*Lilium* spp.‘Manissa’). Flowers were obtained from a local commercial grower (Meilan Florist, Lanzhou, China) and harvested early in the morning. Cut lily flowers were harvested at the tight and green bud (puffy bud) stage (commercial harvest stage) (Zhang et al., 2018a). Buckets containing the flower stems were covered with a plastic film shroud to minimize moisture loss and physical damage during transportation. After reaching the laboratory for approximately 30 min, the stems were cut under water to a length of 60–70 cm, which avoided air embolism, reduced water loss, and maintained the water balance of cut flowers. These flowers were then held in the laboratory. The laboratory was maintained at a temperature of 25 ± 3°C, a relative humidity of 60 ± 5%, and a photon irradiance of 15 μmol m^–2^ s^–1^. The flowers with an identical size, color, and no mechanical damage or infections were selected to observe the postharvest senescence of flowers. Each treatment was carried out in three replicates, with each replication consisting of five flowering stems. The whole flowers (including distal and proximal tepals, stamens, and carpels) were then sampled every 2 days and randomly mixed for E-nose and GC-MS detection (Figure 1).

### Chemicals and Instruments

Chromatographic 2-octanol obtained from Sigma Aldrich (United States) was used as an internal standard for quantitative analysis. SPME fibers and injection handles were purchased from Supelco of the US. The 7890B–7000C GC–MS detector with automatic deconvolution system (AMDIS) and standard mass spectrometry library (NIST 2014) workstation was purchased from the United States Agilent. DB-WAX elastic quartz capillary column (30 m × 0.25 mm, 0.25 μm) was also purchased from the United States Agilent. The 15-ml screw-top headspace bottle with black opening screw cap and silicone septum and constant temperature metal magnetic stirrer are all from Shanghai AnPu Experimental Technology Co., Ltd.

### E-Nose Analysis

The E-nose analysis was conducted according to the procedure described by Li et al. (2017) with modifications. The headspace analysis was performed with a commercial PEN3.5 E-nose (Airsense Analytics, GmBH, Schwerin, Germany). The system contained 10 metal oxide sensors as shown in Supplementary Table 1. Prior to detection, each sample (5 g of flower samples from 5 flowers) was placed in an airtight glass vial and closely capped with a PTFE-silicon stopper. Then, the samples were kept at 25 ± 1°C for approximately 40 min (headspace-generation time). The detection time of the sample was 120 s, the cleaning time of the sensor was 60 s, and the adjustment time of automatic zero was 10 s. All samples were run with three repetitions.

### HS-SPME-GC-MS Analysis

The GC-MS conditions were conducted according to the procedure described by Wei et al. (2021) with some modifications. For that purpose, the SPME fiber used was DVB/CAR/PDMS (50/30 mm) (Supelco, Bellefonte, PA, United States). The lily sample was taken out of liquid nitrogen and quickly ground to the homogenized state, then the homogenate (5 g of flower samples) was added in a 15-ml screw-head headspace vial containing a magnetic stirring rotor and 2 ml ultrapure water to fully stir. Later, a certain amount of NaCl (1 g) and 10 μl of 2-octanol (41.05 mg l^–1^) internal standard were added. The sample vial was then tightened and fitted with a PTFE silicon stopper quickly. Later, the headspace bottle was equilibrated at a certain time (15 min) for a certain temperature (60°C) on a metal heating agitation platform at 500 rpm. Then, the extraction and adsorption were carried out by inserting the pretreated SPME fibers into the headspace bottle for a certain time (30 min) with consecutive heating and agitation. After extraction, the fiber was desorbed into the GC injection port for a certain time (5 min) and then subjected to the GC-MS analysis.

The volatiles were separated on a DB-WAX elastic quartz capillary column (30 m × 0.25 mm, 0.25 μm) with helium (≥99.999% purity) at a flow rate of 1.0 ml min^–1^ as the carrier gas. Splitless injection mode was adopted during volatile insertion at 250°C. The oven temperature program was as follows: initial temperature 40°C (held for 3 min) and increased to 150°C at a rate of 4°C min^–1^, then a ramp of 8°C min^–1^ to 210°C, and held for 3 min, which took 41 min in the entire procedure. The mass spectrometer was operated by the electron impact (EI) method with an ionization energy of 70 eV and a source temperature of 230°C. The acquisition was full-scan mode and mass acquisition range of 33–550 m⋅^–1^. The filament current and the quadrupole temperature were 150 μA and 250°C, respectively.

### Qualitative and Quantitative Analysis of Volatile Compounds

Every composition was analyzed by the computer workstation’s automatic deconvolution system (AMDIS) and mass spectrometry library (NIST 2014) according to its mass fragmentation pattern from the spectral database after the GC-MS analysis. Volatile components with a matching degree greater than 80% were maintained. The concentration of each individual compound in the cut lilies was calculated using the internal standard method, and the formula is as follows: the content of each composition (μg⋅tkg^–1^) = (*A*1/*A*2) × (*M*1/*M*2) × 1,000 (*A*1: the component area of the detected composition; *A*2: the component area of the internal standard; *M*1: the amount of the internal standard; and *M*2: the amount of the sample).

### Calculation of Odor Activity Values

Odor activity values (OAVs) are classical coefficients commonly used in the evaluation of aromatic substances. The calculation formula is as follows: OAVs = *C*_*i*_/*OT*_*i*_ (*C*_*i*_: the actual concentration of a certain volatile in lily samples and *OT*_*i*_: odor olfaction threshold) (Liu et al., 2018). When the OAVs are equal to or greater than 1, components contribute significantly to the overall aroma of cut lilies since they have reached the olfactory threshold of the human nasal cavity, while components with OAVs smaller than 1 may not play a direct role (Zhu et al., 2020a).

### Statistical Analysis

The Excel 2010 software was conducted for statistical analysis and charting of data. The data were analyzed using SPSS 22.0 (SPSS Inc., Chicago, IL, United States). Contents of different components were presented as the mean ± SE of three independent experiments with three replicated measurements. Differences among treatments were analyzed using the Duncan’s multiple range test at a *P* < 0.05 level.

## Results and Discussion

### E-Nose Analysis

#### E-Nose Response to Cut Lily Flowers During the Postharvest Process

**FIGURE 2:**
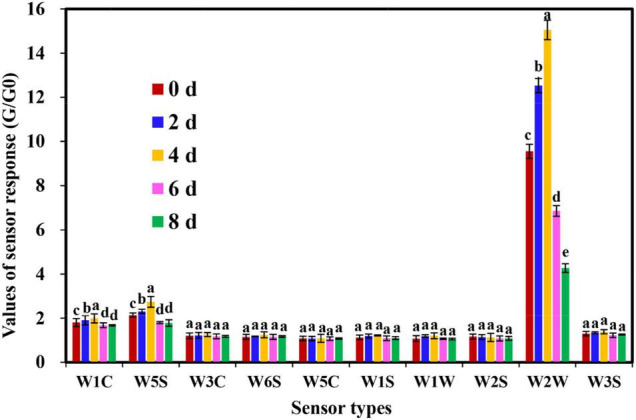
Response values of electronic nose (E-nose) sensors for cut lily flowers during the postharvest process. The values (means ± SE) are the averages of three independent experiments (*n* = 5). Bars not sharing the same letters on the same sensors indicate statistically significant differences using the Duncan’s multiple range test (*P* < 0.05).

In this study, the comprehensive flavor characterization of cut lilies was analyzed using an E-nose equipped with 10 types of sensors. According to Figure 2, the response value of sensors W3C, W6S, W5C, W1S, W1W, W2S, and W3S to all samples was small and almost unchanged. Subsequently, the response value of sensor W2W was the highest, and sensors W1C and W5S were also slightly responded for lilies during the postharvest process (Figure 2). Interestingly, W2W, W1W, and W5C sensors also contributed significantly to the flavor discrimination of shrimp paste during the process of discrimination of shrimp paste (Fan et al., 2017). In addition, the response values of sensors W2W, W1C, and W5S on day 4 were significantly higher than those of the other sensors, and the response of W2W for 4-day samples was significantly different from those of the other samples (Figure 2), implying the emergence of flavor intensity on day 4 after lily harvest. Undeniably, the sensor W2W contributed significantly to the flavor discrimination during the vase period of cut lily flowers.

#### PCA of E-Nose Analysis

**FIGURE 3:**
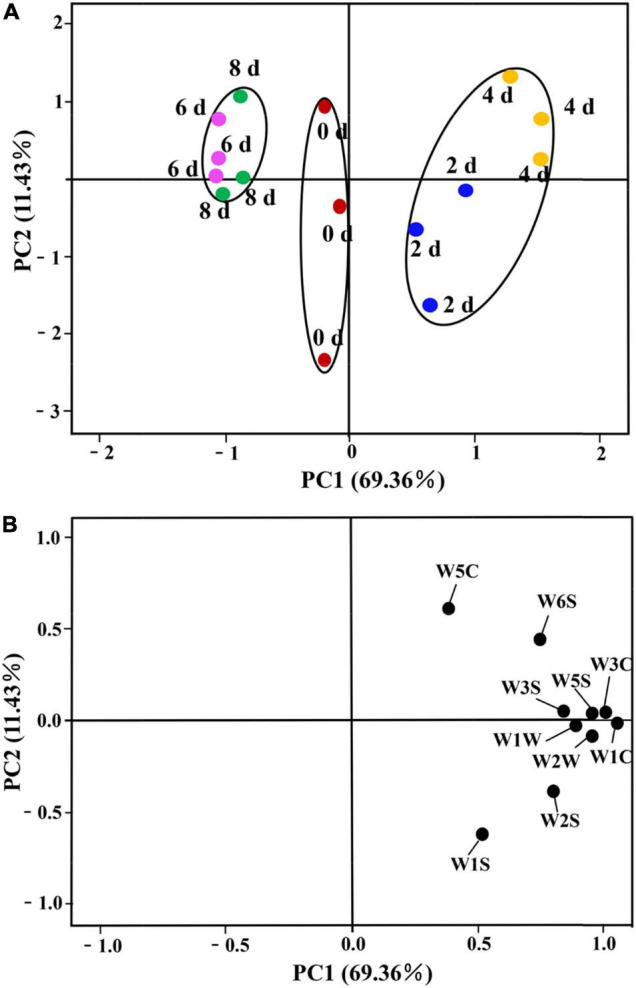
Principal component analysis (PCA) of cut lilies during the postharvest process based on E-nose date. **(A)** Score plot; **(B)** loading plot.

The PCA is a broad statistical procedure by applying an orthogonal transformation to transform a set of observations of possibly correlated variables into a set of values of linearly uncorrelated variables called principal components (PCs). PCA explains the relationship of objects and the correlation structure of the variables (Wei et al., 2021). PCA was performed on the dataset of the response values of E-nose to evaluate the influence of different postharvest times on the grouping of lily samples. The two-dimensional bi-plots of score and loading of the lily samples are presented in Figure 3. The PCs, namely, PC1 and PC2 represented 69.36 and 11.43% of the total variance, respectively, with the cumulative contribution rate of the first two PCs accounting for 80.70%, which indicated that they were sufficient to explain the total variance in the dataset (Dong et al., 2019), and could be used to distinguish lily samples at different vase times. Satisfactorily, it could be observed that the grouping of the samples could be divided into three categories through the first PCs. The lily samples on days 2 and 4 are clustered into one category, being located to the right of the X-axis. The second category is the 6- and 8-day lily samples, which are located to the left of the X-axis. The third category is the 0-day-cut lilies, the day of harvest when the first PC is near 0 (Figure 3A). Furthermore, lily samples on days 2 and 4 could be divided into two categories through the second PCs. In combination with Figure 3B, it can be seen that W5S, W1C, and W2W are significantly positively correlated with the first PC and are the main sensors for distinguishing different samples. Sensors W5C and W1S have the largest positive and negative effects in the second PC, respectively. Thus, the sensor W1S contributed more to the 2-day lily samples, and the response values of W5C might be more correlated with the 4-day lily samples.

#### HCA of E-Nose Analysis

**FIGURE 4:**
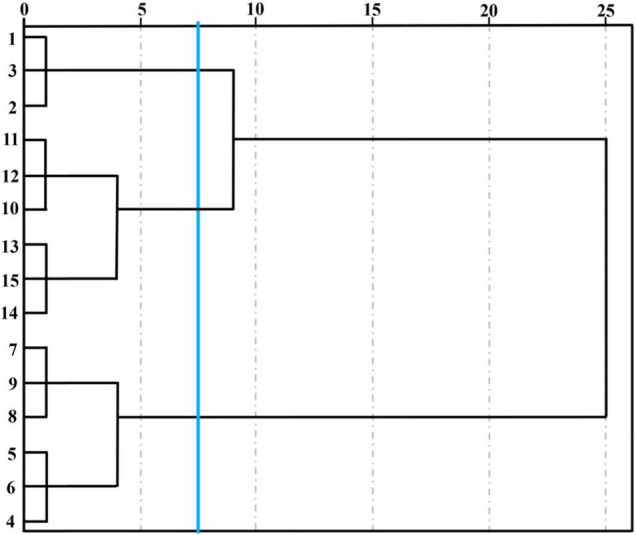
Hierarchical cluster analysis (HCA) of cut lilies during the postharvest process based on E-nose date. Note: cut lilies on day 0 (1, 2, 3), on day 2 (4, 5, 6), on day 4 (7, 8, 9), on day 6 (10, 11, 12) and on day 8 (13, 14, 15).

Unsupervised data processing as a visualization protocol was usually used to evaluate clustering tendency when some indicators were applied to discriminate different samples (Lv et al., 2015). stocktickerHCA was a numerical data consolidation method that could accurately describe the otherness among different cut lily samples during the vase period and was conducive to locate the homogeneity of cut lily flowers to some extent in accordance with similar characteristic volatiles. We referred to the system clustering model characterized through the inter-group connection method with the measurement standard of Euclidean distance employed by Chen et al. (2018). The coalescent data were conducted as a hierarchical dendrogram, where the horizontal axis showed the Euclidean distance among groups and the vertical axis displayed the similarity and diverse degree of flavor in lily flowers. stocktickerHCA can not only judge the repeatability within the group, but more importantly, it can distinguish the differences between different treatments. Three lily samples at each postharvest time were very closely related and could be grouped into a small category (Figure 4), indicating that the E-nose has good reproducibility when evaluating the cut lily aroma at different postharvest times. It could be obtained on the basis of Figure 4, and five different lily flowers were evidently classified into 3 groups at the Euclidean distance of 7.5. The 0-day-cut lilies (1, 2, 3) belonged to one group, the lily samples on days 2 (4, 5, 6) and 4 (7, 8, 9) belonged to one group, and 6-day (10, 11, 12) and 8-day (13, 14, 15) cut lilies belonged to another group. In addition, the relationship between 0-day lilies and 6- and 8-day lilies is relatively close. When the Euclidean distance continues to expand, they could also be clustered into one category, which is completely consistent with the results of PCA. However, the 2- and 4-day lily samples were relatively distant from 0-day-cut lilies. It could be speculated that the main aroma types might change at this time, and the response intensity of sensors W2W and W5S is significantly enhanced. The results of PCA and stocktickerHCA in distinguishing the aroma of lily flowers at different vase times are completely consistent, indicating that the combination of the two methods is feasible and accurate in detecting the flavor changes during the vase period of cut lilies with E-nose. This can not only identify the type of characteristic sensor but also distinguish and categorize cut lily flowers during the vase period.

### HS-SPME-GC-MS Analysis

#### The Differences in Volatile Compounds of Cut Lily Flowers During the Postharvest Process

The volatile profile of cut flowers constitutes a very important organoleptic characteristic for the distinguishing and application of cut flowers during the vase period. To evaluate the flavor change of cut lily flowers after postharvest, volatile compounds were monitored using HS-SPME/GC–MS to provide the volatile profile of each sample at different postharvest times. In this study, a total of 59 different volatile compounds were detected in cut lily flowers. The volatile compounds were classified into 8 groups based on their chemical properties, including 10 aldehydes, 14 alcohols, 13 esters, 6 phenols, 5 hydrocarbons, 2 ketones, 5 acids, and 4 others (Table 1). During the *Tremella aurantialba* fermentation, the identified compounds consisted of 5 families, 10 aldehydes, 7 esters, 8 alcohols, 2 ketones, and 16 heterocyclic and aromatic compounds (Dai et al., 2018), resulting in a predominant flavor during the *T. aurantialba* fermentation. Moreover, Yin et al. (2015) reviewed that volatile compounds of water lily flowers comprised several classes, which was in accordance with this study, thereby implying the diversity of volatile substances of lilies. Approximately 40 volatile compounds from flowers reported in the literature (Ike Da et al., 2010; Gaytán et al., 2013; Yang et al., 2017) could also be authenticated in our samples. Approximately 10 kinds of aroma compounds were identified for the first time in cut lilies, which presumably attributed to many other factors, including genotype, cultivation region, harvest maturity, and cultivar differences. However, some of these compounds had been reported in cabbages (Wei et al., 2021), grapes (Wang et al., 2017), and Chinese jujubes (Chen et al., 2018).

**TABLE 1:** The composition and content of volatile compounds in cut lily during the postharvest process by headspace solid-phase microextraction gas chromatography-mass spectrometry (HS-SPME/GC–MS) methodology.

Group (No.)	Volatile compound*^a^*	The content of volatile compound (μg kg^–1^)^b^					MS match index (%)	Molecular formula	CAS
		0 day	2 days	4 days	6 days	8 days			
**Aldehyde**									
1	(E)-2-hexenal	1119.96 ± 82.87a	698.39 ± 29.62b	845.09 ± 80.35b	201.35 ± 29.72c	186.34 ± 19.08c	85.59	C_6_H_10_O	6728-26-3
2	Hexanal	341.41 ± 8.46a	151.57 ± 11.90b	215.65 ± 12.59b	103.32 ± 8.02d	122.54 ± 1.11cd	89.00	C_6_H_12_O	66-25-1
3	(E)-non-2-enal	220.24 ± 32.0a	145.40 ± 17.12b	72.16 ± 2.9c	94.76 ± 19.18bc	–	91.05	C_9_H_16_O	18829-56-6
4	(Z)-tetradec-9-enal	159.19 ± 5.56a	–	10.70 ± 0.51b	–	18.01 ± 0.48b	87.26	C_14_H_26_O	53939-27-8
5	(E)-2-Octenal	145.96 ± 24.50a	37.57 ± 4.91b	42.03 ± 1.25b	45.12 ± 1.25b	53.12 ± 14.85b	88.74	C_8_H_14_O	2548-87-0
6	(E)-2,4-Heptadienal	120.13 ± 13.91a	16.10 ± 0.85b	14.38 ± 1.92b	–	–	85.70	C_7_H_10_O	4313-03-5
7	(Z)-9-Hexadecenal	92.63 ± 5.31b	497.07 ± 0.36a	469.67 ± 14.20a	91.69 ± 13.73b	102.12 ± 14.46b	91.61	C_16_H_30_O	56219-04-6
8	Benzeneacetaldehyde	68.05 ± 10.33a	41.73 ± 6.01b	41.32 ± 4.11b	58.72 ± 5.60ab	58.29 ± 10.57ab	83.28	C_8_H_8_O	122-78-1
9	Non-anal	63.91 ± 9.45a	76.87 ± 8.52a	76.81 ± 8.52a	64.11 ± 4.93a	70.57 ± 3.57a	94.29	C_7_H_6_O	124-19-6
10	Vanillin	31.70a	–	–	–	–	85.41	C_11_H_20_O	121-33-5
**Alcohols**									
11	Linalool	861.36 ± 50.86b	892.61 ± 53.05b	1184.63 ± 17.24a	317.89 ± 58.93c	204.75 ± 58.93c	93.1	C_10_H_18_O	78-70-6
12	Alpha-Terpineol	254.57 ± 35.33c	379.16 ± 9.24b	555.53 ± 69.67a	227.73 ± 27.82c	202.48 ± 49.87c	83.38	C_10_H_18_O	10482-56-1
13	(E)-hex-3-en-1-ol	91.32 ± 1.83a	51.14 ± 0.75b	47.75 ± 4.75b	–	–	85.99	C_6_H_12_O	928-97-2
14	Geraniol	90.8 ± 15.38c	129.96 ± 1.9ab	164.02 ± 5.51a	122.78 ± 3.63bc	113.35 ± 20.32bc	83.72	C_10_H_18_O	106-24-1
15	Eucalyptol	87.11 ± 4.53b	188.69 ± 2.84a	179.97 ± 2.84a	9.54 ± 0.55c	–	90.61	C_10_H_18_O	470-82-6
16	(E)-2-hexen-1-ol	81.53a	–	–	–	–	80.62	C_10_H_18_O	928-95-0
17	Farnesol	40.31a	–	–	–	–	85.94	C_6_H_12_O	4602-84-0
18	Terpinen-4-ol	27.16 ± 0.14c	107.57 ± 4.48a	101.75 ± 8.76a	29.28 ± 8.04c	64.54 ± 5.88a	85.27	C_15_H_26_O	562-74-3
19	Geranyl Geraniol	8.16 ± 0.46a	–	60.97 ± 1.47b	20.26 ± 1.8c	12.82 ± 0.21d	87.57	C_20_H_34_O	24034-73-9
20	Nerol	–	135.42 ± 2.82b	189.88 ± 1.86a	43.62 ± 4.08c	26.63 ± 2.88b	87.6	C_10_H_18_O	106-25-2
21	5-Indanol	–	119.33a	–	–	–	87.75	C_9_H_10_O	1470-94-6
22	P-menth-1-en-8-ol	–	17.05a	–	–	–	81.76	C_10_H_18_O	98-55-5
23	Benzyl alcohol	–	–	–	70.86 ± b	103.41 ± c	92.29	C_7_H_8_O	100-51-6
24	5-Indanol	–	–	82.43a	–	–	86.22	C_9_H_10_O	1470-94-6
**Esters**									
25	2-Hydroxybenzoic acid phenylmethyl ester	278.52 ± 34.11b	392.30 ± 35.03a	680.24 ± 112.58a	181.76 ± 13.7bc	59.03 ± 10.87c	90.77	C_14_H_12_O_3_	118-58-1
26	Methyl salicylate	222.29a	–	–	–	–	87.52	C_8_H_8_O_3_	119-36-8
27	Ethyl 9-oxononanoate	133.19 ± 3.63a	101.47 ± 3.66ab	76.50 ± 2.04b	72.73 ± 1.23c	34.55 ± 8.17c	92.3	C_11_H_20_O_3_	3433-16-7
28	Benzyl benzoate	118.92 ± 3.52c	563.54 ± 35.03b	909.55 ± 82.67a	78.74 ± 5.34c	58.69 ± 4.19c	95.36	C_14_H_12_O_2_	120-51-4
29	Ethyl Palmitate	38.68 ± 3.24e	204.55 ± 5.26b	224.47 ± 7.61a	119.8 ± 1.38c	88.54 ± 2.18d	89.49	C_18_H_36_O_2_	628-97-7
30	Ethyl benzoate	–	61.32 ± 3.67a	17.42 ± 2.26b	–	–	92.01	C_9_H_10_O_2_	93-89-0
31	Benzoic acid, 2-phenylethyl ester	–	119.01 ± 3.61b	173.78 ± 6.88a	11.68 ± 0.74c	–	96.59	C_15_H_14_O_2_	94-47-3
32	Ethyl myristate	–	101.09 ± 4.53a	69.71 ± 2.45b	70.15 ± 9.90b	56.81 ± 1.58b	80.52	C_16_H_32_O_2_	124-06-1
33	Ethyl laurate	–	43.25 ± 1.08a	23.19 ± 2.54b	16.54 ± 2.52b	11.88 ± 0.92b	87.91	C_14_H_28_O_2_	106-33-2
34	Methyl palmitoleate	–	–	38.04a	–	–	80.51	C_17_H_32_O_2_	1120-25-8
35	Ethyl Oleate	–	–	28.92 ± 0.39	13.65 ± 0.26	23.29 ± 2.09	80.79	C_20_H_38_O_2_	111-62-6
36	Isopropyl myristate	–	8.74 ± 0.46a	22.65 ± 6.46a	10.98 ± 1.66a	–	81.52	C_17_H_34_O_2_	110-27-0
37	Benzyl oleate	–	22.81 ± 2.18b	82.23 ± 4.81a	11.71 ± 1.84b	–	87.06	C_25_H_40_O_2_	55130-16-0
**Phenolic**									
38	P-Cresol	317.3 ± 9.79b	280.65 ± 5.065b	438.53 ± 26.94a	62.05 ± 1.64c	24.24 ± 2.06c	96.72	C_7_H_8_O	106-44-5
39	3-Hydroxy-4-methoxytoluene	309.25 ± 4.02c	417.48 ± 7.38b	594.88 ± 8.14a	101.04 ± 2.07d	45.29 ± 0.84e	96.35	C_8_H_10_O_2_	1195-09-1
40	Methyleugenol	95.46 ± 2.86a		20.44 ± 0.73b	–	–	865.43	C_11_H_14_O_2_	93-15-2
41	2-methoxy-5-(prop-2-en-1-yl)phenol	89.40 ± 3.42b	144.88 ± 6.67a	138.04 ± 2.70a	–	–	89.93	C_10_H_12_O_2_	501-19-9
42	Eugenol	–	137.08 ± 4.52a	186.04 ± 3.12b	41.20 ± 3.4a	–	90.89	C_10_H_12_O_2_	97-53-0
43	p-allylphenol	–	–	43.22a	–	–	88.79	C_9_H_10_O	501-92-8
**Hydrocarbons**									
44	Pentalene, octahydro-2,5-dimethyl-	45.46 ± 1.82a	–	24.65 ± 0.11b	–	–	86.88	C_10_H_18_	28588-55-8
45	Pentadecane	29.03 ± 0.33b	–	–	–	52.66 ± 8.44a	83.7	C_15_H_32_	629-62-9
46	1H-Indene, 5-butyl-6-hexyloctahydro-	16.09 ± 2.49a	16.42 ± 3.82a	22.24 ± 0.11a	–	–	81.77	C_19_H_36_	55044-36-5
47	D-Limonene	15.30 ± 0.06bc	14.35 ± 2.01bc	12.08 ± 2.01c	34.46 ± 2.70b	39.46 ± 2.70a	87.45	C_10_H_16_	5989-27-5
48	2,6,10-trimethyltetradecane	–	49.16 ± 6.83a	40.44 ± 1.83a	14.55 ± 0.252b	20.38 ± 1.21b	87.85	C_17_H_36_	14905-56-7
**Ketone**									
49	Geranyl Acetane	66.89 ± 13.97b	422.38 ± 30.01a	498.22 ± 21.77a	445.75 ± 22.02a	447.31 ± 23.58a	86.89	C_13_H_22_O	3796-70-1
50	6-Methyl-5-hepten-2-one		242.34 ± 20.97b	249.18 ± 18.60b	656.85 ± 32.02a	127.35 ± 15.95c	84.02	C8H14O	110-93-0
51	Oleic acid	13.26 ± 0.20b	24.26 ± 0.20a	22.52 ± 1.69a	11.99 ± 0.14b	–	88.76	C_18_H_34_O_2_	112-80-1
52	Linolic acid	22.27 ± 0.14a	–	21.94 ± 0.04a	17.18 ± 0.24b	–	81.09	C_18_H_32_O_2_	60-33-3
53	myristoleic acid	–	–	14.48 ± 4.14a	–	–	80.32	C_18_H_34_O_2_	544-64-9
54	Non-anoic acid	–	–	–	48.44 ± 2.04a	–	80.88	C_10_H_18_O_3_	1931-63-1
55	p-(benzyloxy)benzoic acid	–	–	–	–	44.1 ± 1.24a	85.32	C_14_H_12_O_3_	1486-51-7
**Others**									
56	2,3-Dimethoxytoluene	23.432 ± 1.67d	586.88 ± 77.32a	435.19 ± 45.0b	622.88 ± 5.26a	126.11 ± 19.06c	87.65	C_9_H_12_O_2_	4463-33-6
57	Oxime-, methoxy-phenyl-_	31.7643 ± 1.67d	96.94 ± 1.07d	216.94 ± 3.32b	391.28 ± 6.47a	136.14 ± 2.47c	83.07	C_8_H_9_NO_2_	1000222-86-6
58	Benzene, 1,2-dimethoxy-4-(1-propenyl)	96.94 ± 8.67d	216.94 ± 19.74c	391.28 ± 6.57a	236.14 ± 11.62d	283.35 ± 19.98b	92.09	C_11_H_14_O_2_	93-16-3
59	Indole	28.8383 ± 1.17c	–	84.64 ± 6.11b	188.54 ± 3.24a	–	93.06	C_8_H_7_N	120-72-9
	**Total**	**5897.79**	**7953.47**	**10156.39**	**4961.12**	**3018.15**			
	**Numbers**	**39**	**40**	**49**	**39**	**32**			

*^a^Volatile compoundhe fruity fragrances of lils detected were integrated with the GC-MS automatic deconvolution system and compared with the standard mass spectrum in the NIST 14 library (MS match index ≥ 80% were listed).*

*^b^Each value is the mean of triplicate biological samples taken from the same cabbage cultivar; “-,” not detected.*

According to Table 1, it was clear that 22 compounds were detected on all days. Among 22 volatile compounds, both (E)-2-hexenal and linalool accounted for approximately 10% of the total content, occupying the most abundant of lilies. Linalool was found as a prominent constituent of the floral scent in *Magnolia kobus* (Azuma et al., 2001) and *Clarkia breweri* (Pichersky et al., 1994). Moreover, Dhandapani et al. (2017) identified that linalool synthase was essential for the production of major volatile organic compounds of champak flowers by metabolite-guided transcriptomics. Thus, this implies that linalool could be a key substance for the floral fragrance of cut lilies, which was also reported in the previous literature (Du et al., 2019; Melo et al., 2021).

Four volatile compounds only existed in 0-day-cut lilies after harvest (Table 1). It is worth mentioning that (E)-2-hexen-1-ol was reported exclusively in lilies by our group. Vanillin, farnesol, and methyl salicylate were identified previously in honeysuckle flowers (Ike Da et al., 2010) and *Hybrid Lilium* (Johnson et al., 2016). Previously, vanillin was widely used in edible spices, which could be found in vanilla seeds (Dong et al., 2014) and *H. Lilium* petals (Johnson et al., 2016). Interestingly, in a recent study, vanillin has been found to have appetite-enhancing effects on vanilla flavors (Ogawa et al., 2018). Since farnesol acts as a solvent to make the fragrance stronger and more uniform when it is decomposed, it is usually used to enhance the fragrance of floral perfumes or aromatherapy oils (Chen and Viljoen, 2010).

Volatile compounds of 5-indanol and p-menth-1-en-8-ol were only identified in cut lilies on day 2. Previously, Johnson et al. (2016) found that *Lilium* cv. ‘Conca d’Or’ accumulated the most indole and the lowest 5-indanol unlike ‘Robina’, which has the inverse accumulation profile, implying that the content of nitrogen-containing volatile compounds in lilies in different varieties or at different postharvest times is different. On day 4, 5 volatile compounds existed in cut lilies (Table 1), among them, 3-methyl-1, 6-heptadien-3-ol, and p-allylphenol were first identified in flower petals, and methyl palmitoleate was previously found in honeysuckle (*Lonicera japonica* Thunb.) flowers (Ike Da et al., 2010).

Non-anoic acid was found in only 6-day-cut lilies (Table 1), which were also identified in *Cymbidium* sp. (Gaytán et al., 2013). Moreover, nonanoic acid, as a main volatile compound, was found in ten different varieties of Chinese jujubes (Chen et al., 2018). Furthermore, another acid, p-(benzyloxy) benzoic acid, only existed in 8-day-cut lily flowers (Table 1). A recent study revealed a new mechanism of acid for the promotion of cell senescence (Chen et al., 2021), which indicated that cut lilies on day 8 after harvest gradually showed a state of aging. These results further indicated the difference and diversity of volatile compounds during the vase period of cut lily flowers.

#### The Tendency of Amount and Content of Volatile Compounds in Cut Lily Flowers During the Postharvest Process

**FIGURE 5:**
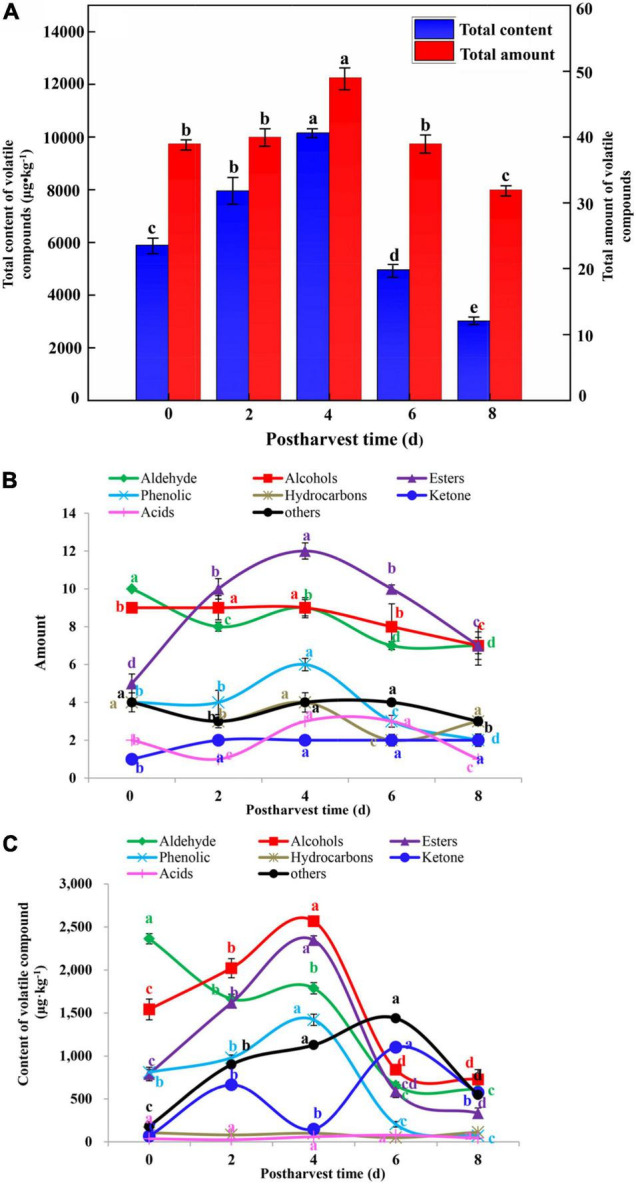
Analysis of the total content and amount **(A)** of volatile compounds of cut lily flowers and the amount **(B)** and content **(C)** of various category volatile compounds of cut lily flowers and during the postharvest process. Different lowercase letters in the figure indicate significant differences at the level of *P* < *0.05*.

From a quantitative perspective, the total content of volatiles identified in cut lilies were analyzed, ranging from 5,897.79 (0 day), 7,953.47 (2 days), 10,156.39 (4 days), 4,961.12 (6 days) to 3,018.15 μg kg^–1^ (8 days) (Figure 5A). Approximately 39, 40, 49, 39, and 32 kinds of volatile compounds were identified in cut lily flowers at 0, 2, 4, 6, and 8 days, respectively (Figure 5A). The amount of alcohols, phenols, and esters first increased and then decreased, and the amount of these volatile compounds reached the highest on day 4. Interestingly, the amount of aldehydes and acids first declined, then rose, and then fell (Figure 5B). That is to say, cut lilies during the postharvest process might exhibit significantly different kinds and amounts of aroma compounds, which might be partly dependent on the growth period (Zhang et al., 2019).

During the vase period of lily, alcohol compounds that contributed to the aroma of cut lilies over 20% (25.41% on day 2 and 25.53% on day 4) were the most abundant in cut lily flowers. Among 8 alcohol compounds, 46.1 and 21.6% of the compounds were linalool and alpha-terpineol on day 4, respectively, which existed on all days and were the predominant volatile compounds in alcohols. Linalool and alpha-terpineol were also identified in other flower petals, such as *Magnolia champaca* (Dhandapani et al., 2017), fresh flowers of *Cymbidium* sp. (Gaytán et al., 2013), and *Lavandula angustifolia* (Smigielski et al., 2018). In addition, linalool and alpha-terpineol have been also identified as the main volatile components of essential oils of lavender flowers (Smigielski et al., 2018). Thus, we speculated that 4-day-cut lilies have the main volatile components of essential oils, which might be suitable for producing lily essential oil. The total content of aldehydes gradually decreased during the postharvest process, ranging from 0 day (2,363.18 μg kg^–1^) to 8 days (610.99 μg kg^–1^) (Figure 5C). This may be due to aldehydes that were converted into alcohols by dehydrogenase (Zhou et al., 2019). Among 10 aldehyde compounds, (E)-2-hexenal had the highest content, accounting for 46% of the total content of aldehydes, which were the main aroma components with contributions that over 47.4% on day 0. It is noteworthy that (E)-non-2-enal, (Z)-tetradec-9-enal, (E)-2-octenal, and (Z)-9-hexadecenal have not been reported in flowers before.

Esters synthesized through the oxidation of fatty acids (Chen et al., 2018) contribute to fruit and floral flavor. In our study, cut lilies on day 4 had the highest total content of esters (2,346.70 μg kg^–1^). Additionally, 2-hydroxybenzoic acid phenylmethyl ester and benzyl benzoate were the main compounds of esters and had the highest contents on day 4 (680.24 and 909.55 μg kg^–1^, respectively) (Figure 5C and Table 1), which was also identified from honeysuckle flowers in previous studies (Ike Da et al., 2010). Similarly, the total content of phenols showed the highest value (1,421.15 μg⋅kg^–1^) on day 4 (Figure 5C), and p-cresol and 3-hydroxy-4-methoxytoluene were the main compounds of phenols in lilies, which were also reported in the previous study (Johnson et al., 2016). Edible flowers are rich sources of phenolic compounds with antioxidants, DPPH radical-scavenging activity, and reducing power. Thus, this implies that 4-day lily flowers might have strong antioxidant capacity and flavor quality. Notably, 4-day-cut lily flowers might facilitate the extraction of phenols.

Ketone is one of the key aroma compounds for flavor. Although there were few amounts of ketones, it had higher contents in cut lily flowers on days 4 (747.40 μg kg^–1^) and 6 (1,102.60 μg kg^–1^) (Figure 5C and Table 1). Geranyl acetone and 6-methyl-5-hepten-2-one were the main compounds of ketones with contributions that were over 13%. In our study, acids showed lower contents (0.3–1.5%) (Figure 5C), and oleic acid and linoleic acid were first reported in this study. Since some alcohols and aldehydes undergo oxidation and other changes to form acid, the content of acid will gradually increase with the decrease of aldehydes and alcohols on days 6 and 8 after lily harvest. Therefore, cut lilies from different vase times might exhibit various kinds and quantities of the volatiles, leading to flavor change during the postharvest process. Thus, cut lily flowers on the 4th day of the vase could exhibit excellent flavor and ornamental quality.

#### Odor Activity Value Analysis and Radar Fingerprint Chart of Volatile Compounds in Cut Lilies During the Postharvest Process

**FIGURE 6:**
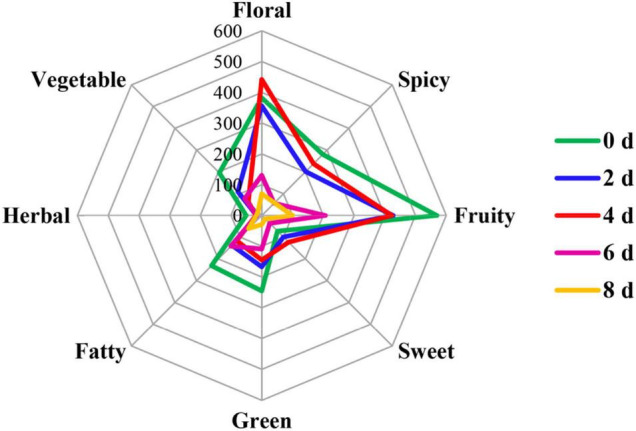
Radar fingerprint chart of the 19 most potent volatile compounds in cut lily flowers during the postharvest process.

Odor activity values refer to the ratio of the actual concentration of volatile compounds in the matrix to its odor threshold, which means the contribution of a volatile to the comprehensive flavor (Zhu et al., 2020b). Usually, when its OAVs are greater than 1, volatile components are considered to make a practical contribution. In our study, there were 19 volatiles with the OAVs over 1 that made contributions to the cut lily’s flavor, which were divided into eight categories of aroma, including floral, pungent, fruity, fatty, sweet, herbal, and vegetable (Table 2). The radar fingerprint chart (RFC) composed of cut lily flowers during the postharvest process is depicted in Figure 6, which makes the changes in the comprehensive odor of cut lilies clearer. The floral and fruity odors were the strongest scent, followed by the spicy odor. A previous study indicated that lily accessions were classified into six groups according to the composition of major scent components: faint-scented, fruity, cool, musky, fruity-honey, and lily (Du et al., 2019). In our study, the floral and fruity odors of cut lilies were richer, mainly including the floral of clove, carnation, rosy, and narcissus, and fruity of apples, oranges, melons, and citrus (Table 2), which make great contributions to the aroma of lilies.

**TABLE 2:** Odor activity values (OAVs) of the 19 most potent volatile compounds in cut lilies during the postharvest process.

Group (No.)^a^	Volatile compound	Odor threshold (*OT*_*i*_)	Odor activity values (OAVs)^c^					Odor description^d^
		μg/kg^b^	0 day	2 days	4 days	6 days	8 days	
**Aldehyde**								
1	(E)-2-hexenal	4	279.99	174.60	211.27	50.34	46.59	Spicy, clove, carnation, fruity
2	Hexanal	75	4.55	2.02	2.88	1.38	1.63	Fresh green, fatty, fruity
3	(E)-non-2-enal	1.5	146.83	96.93	48.11	63.17	–	Fatty, green, cucumber, citrus
5	(E)-2-Octenal	3	48.65	12.52	14.01	15.04	17.71	Fresh, cucumber, fatty, green, herbal, banana
8	Benzeneacetaldehyde	25	2.72	1.67	1.65	2.35	2.33	Green, sweet, rosy, fruity
9	Non-anal	2.5	25.56	30.75	30.75	25.64	28.23	Rosy, fresh, fatty,
**Alcohols**								
11	Linalool	15	57.42	59.51	78.98	21.19	13.65	Citrus, orange, floral, sweet, rose
14	Geraniol	20	4.54	6.50	8.20	6.14	5.67	Sweet, floral, rosy, fruity, fatty
15	Eucalyptol	64	1.36	2.95	2.81	0.15	–	Sweet, fresh, herbal
19	Geranyl Geraniol	40	0.20	–	1.52	0.51	0.32	Green, leafy, floral
**Esters**								
25	2-Hydroxybenzoic acid phenylmethyl ester	82	0.68	0.96	1.66	0.44	0.14	Floral, herbal, sweet, balsam
26	Methyl salicylate	40	5.56	–	–	–	–	Green, sweet
37	Benzyl oleate	22	–	1.04	3.74	0.53	–	Sweet, waxy, floral
**Phenolic**								
38	P-Cresol	25	12.69	11.23	17.54	2.48	0.97	Narcissus, green, floral
39	3-Hydroxy-4-methoxytoluene	13	23.79	32.11	45.76	7.77	3.48	Fruity, green, floral
42	Eugenol	7.1	–	19.31	26.20	5.80	–	Sweet, spicy, clove
**Ketone**								
49	Geranyl Acetane	60	1.11	7.04	8.30	7.43	7.46	Fresh, green, fruity, waxy, rose
50	6-Methyl-5-hepten-2-one	68	–	3.56	3.66	9.66	1.87	Citrus, green, apple,
**Others**								
59	Indole	23	–	3.68	8.20	–	–	Floral

*^a^The serial number of the volatile compounds are consistent with Table 1.*

*^b^The odor thresholds of volatile compounds were obtained from the following report (van Gemert, 2003; George, 2010; Pino and Mesa, 2010).*

*^c^OAVs means the ratio of its actual concentration of a volatile in cut lilies to its odor threshold; OAVs = C_i_/OT_i_ (C_i_: the actual concentration of a certain volatile in lily samples; OT_i_: odor olfaction threshold); “-,” not detected.*

*^d^ Odor descriptions were adapted from the online database (http://www.thegoodscentscompany.com).*

The floral odor first strengthened and then gradually weakened, reaching its strongest scent on day 4 (Figure 6). Due to a lower threshold of (E)-2-hexenal (4 μg kg^–1^) and its high concentration, it contributed greatly to the fruity and floral aromas of cut lilies with the OAVs above 200 on day 4. Under the action of alcohol dehydrogenase, aldehyde substrates were metabolized into alcohols, which were used as direct substrates to participate in the synthesis of ester aroma substances, then promoting the fragrance emission of volatile compounds (León-Sánchez et al., 2009). That may be the main reason why 4-day-cut lilies were stronger than 0- and 2-day-cut lilies in floral aroma intensity. However, OAVs of (E)-2-hexenal were between 1 and 7 in green cabbages (Wei et al., 2021), which may be due to the different activity and contribution of volatile compounds on different species. Subsequently, linalool also had a great contribution to the floral aroma of lilies and had the highest OAV (78.98 μg kg^–1^) on day 4. A previous study showed that (E)-2-hexenal had a negative correlation with the aroma quality of black tea, and the ratio of linalool to (E)-2-hexenal could be used to judge the quality of black tea aroma (Fernando and Roberts, 2010). That is to say, in our study, linalool with higher OAV and (E)-2-hexenal with lower OAV on day 4 might contribute greatly to lily flavor.

The fruity fragrances of lilies gradually weakened during the vase period of cut lilies (Figure 6). Wang et al. (2017) suggested that drying or thermal treatment could induce changes in some novel volatile substances and then change flavor quality. Thus, the weakness of the fruity flavor of lilies might be due to the water loss-induced drying after the postharvest process. In addition to (E)-2-hexenal, linalool, and 3-hydroxy-4-methoxytoluene, (E)-non-2-enal with a threshold value of 1.5 μg kg^–1^ significantly contributed to the fruity flavor. Notably, ketones (geranyl acetone and 6-methyl-5-hepten-2-one) with OAVs greater than 1 also contributed greatly to the fragrance of cut lilies (Table 2). Previously, 6-methyl-5-hepten-2-one had also been found in sun-dried raisins, whereas its OAVs were less than 1, thereby hardly contributing to the aroma of raisins (Wang et al., 2017). Interestingly, Smigielski et al. (2018) indicated that the essential oil from fresh lavender flowers received the highest marks among the distinguishing features: floral, fresh, and green. This implies that the floral and fruity might be the main odors for essential oil isolated from lily flowers, which needs to be further explored. Thus, the potential use of lily oil as cosmetics and food additives might be an important research topic in the future.

It is worth noting that spicy odor was a stronger scent of cut lilies, mainly including (E)-2-hexenal with higher OAVs and phenols of eugenol with lower OAVs. Eugenol was also identified in lily bulbs as a clove-like compound with a lower OAV (Chiang, 2016). Recently, Wei et al. (2021) indicated that the spicy odor played an indispensable role in the fragrance of cabbages and might be related to the regulation of the anthocyanin metabolism pathway. The green aroma was mainly comprised of hexanal, (E)-non-2-enal, and P-cresol and gradually weakened during the postharvest process (Figure 6), which might be the main reason why cut lilies were weaker on day 8 than those on other days. In addition, cut lilies after harvest also exhibited fatty, sweet, herbal, and vegetable aromas, which were low-intensity aromas relatively and were not toilless to be smelled in cut lilies for the masking effect when the floral, fruity, and pungent odors were strong. Thus, the overall OAVs of cut lilies on day 4 were universally much higher than those on other days, and the floral odor was the strongest scent, which hypothesized that the activities of related metabolic enzymes and the content of volatile precursors in cut lilies on day 4 were relatively higher.

#### PCA of HS-SPME-GC-MS Analysis

**FIGURE 7:**
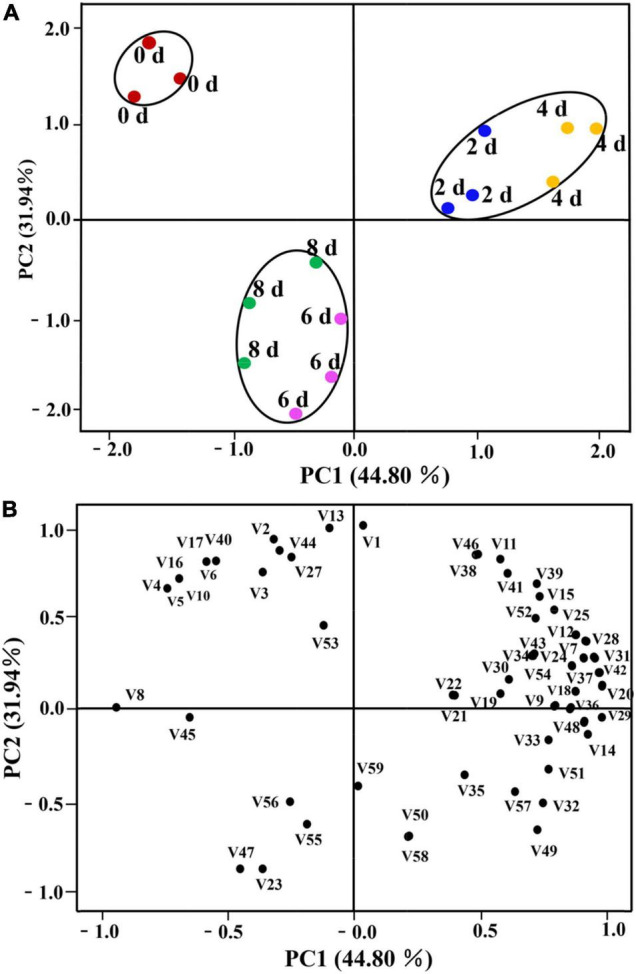
Principal component analysis of cut lilies in different days after harvest and 59 volatile compounds. **(A)** Score plot; **(B)** loading plot. The number code after “V” in (7B) also corresponds to the volatile code in Table 1.

The PCA was performed to observe possible sample distribution according to different postharvest times of cut lilies. The PCA plot presented in Figures 7A,B explained 76.74% of the total cumulative variance contribution. PC1 and PC2 accounted for 44.80 and 31.94% of the total variance, respectively, as shown in the score plot of PCA (Figure 7A). Similar to the results of the PCA of the E-nose, all lily samples in different postharvest times were clustered into three distinct groups in the dimensional graph based on the PC1 × PC2 score. The 0-day-cut lily samples were all located in the second quadrant of the graph, whereas samples on days 2 and 4 were in the first quadrant, and 6- and 8-day lily samples were localized in the third quadrant of the graph. Furthermore, Figure 7B shows the relationship of lily samples in different postharvest times with volatile compounds. It could be observed that volatile substances, namely, benzeneacetaldehyde (V8), pentadecane (V45), nerol (V20), and ethyl palmitate (V29) are highly and positively related to PC1, whereas (E)-2-hexenal (V1), (E)-hex-3-en-1-ol (V13), D-Limonene (V47), and benzyl alcohol (V23) have more contribution in PC2. In addition, it could also be found that unique volatile compounds of vanillin (V10), (E)-2-hexen-1-ol (V16), and farnesol (V17) and higher content compounds of (Z)-tetradec-9-enal (V4), (E)-2-Octenal (V5), and methyleugenol (V40) mainly distinguished 0 day-lily samples from other samples. The volatile compounds that contributed greatly to the separation of lily flowers on days 2 and 4 were benzyl benzoate (V28), benzoic acid, 2-phenylethyl ester (V31), and eugenol (V42). Finally, 6- and 8-day samples were characterized by benzyl alcohol (V23) and D-limonene (V47), which were correlated with PC1.

#### HCA of HS-SPME-GC-MS Analysis

**FIGURE 8:**
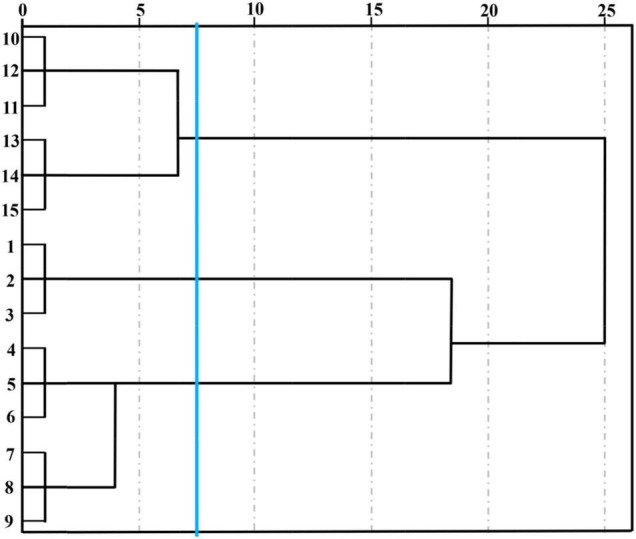
Hierarchical cluster analysis of cut lily flowers during the postharvest process based on the composition and content of their volatile compounds. Note: cut lilies on day 0 (1, 2, 3), on day 2 (4, 5, 6), on day 4 (7, 8, 9), on day 6 (10, 11, 12), and on day 8 (13, 14, 15).

The 59 kinds of volatile compounds and corresponding contents were the elements of this new data matrix, and the matrix with dimensions of 5 samples × 59 variables (volatiles) was constructed to perform HCA (Figure 8). The Euclidean distance of the GC-MS cluster map classification was consistent with the E-nose at 7.5. It could be observed from Figure 8 that cut lilies were evidently classified into 3 groups. Cut lilies on day 0 belonged to one group, 2- and 4-day lilies belonged to one group, and 6- and 8-day samples belonged to one another group. These classification groups were consistent with the PCA results, in which all lily samples were fully distinguished according to their vase time. In addition, three replicate samples at each postharvest time were closely related, which further proved the great repeatability within the group. Interestingly, the clustering results of GC-MS were also consistent with the E-nose. Therefore, it could be speculated that the E-nose and GC-MS are highly correlated in distinguishing the aroma changes of lily flowers during the postharvest process, which could be confirmed by PCA and HCA. In conclusion, the E-nose combined with GC-MS applied to the dynamic profile analysis of cut flower aroma was matching and corresponding, and it was highly efficient and meaningful in the flavor research and characteristic substance screening of cut flowers.

## Conclusion

The volatile profile of cut lilies during the vase period was identified by HS-SPME-GC-MS coupled with E-nose. E-nose analysis found that the response values of sensors W2W, W1C, and W5S were significantly higher than those of the other sensors on day 4, implying the emergence of flavor intensity on day 4 after lily harvest. A total of 59 volatiles were identified and quantified in cut lilies using HS-SPME-GC-MS methods, mainly including alcohols, aldehydes, esters, and phenols. The total content of volatiles was ranging from 5,897.79 (0 day), 7,953.47 (2 days), 10,156.39 (4 days), 4,961.12 (6 days) to 3,018.15 μg⋅kg^–1^ (8 days). Approximately 39, 40, 49, 39, and 32 kinds of volatile compounds were identified in lily flowers at 0, 2, 4, 6, and 8 days, respectively. Among them, there were 19 characteristic volatiles (OAVs > 1), making large contributions to the lily flavor. Floral and fruity aromas in lilies were stronger, followed by pungent aromas. Furthermore, E-nose and HS-SPME-GC-MS are highly correlated in distinguishing the aroma changes of lily flowers during the postharvest process, which could be confirmed by PCA and HCA. In summary, cut lily flowers on the 4th day of the vase might exhibit excellent flavor and aroma quality taking into consideration of the flavor intensity and diversity. These give a new insight into the mechanisms of flavor change and establish theoretical guidance for commercial application of cut flowers and extraction of characteristic volatiles during postharvest periods. Thus, further investigations are required focusing on the metabolic regulation of characteristic volatiles during the vase period of cut lily flowers.

## Data Availability Statement

The original contributions presented in this study are included in the article/Supplementary Material, further inquiries can be directed to the corresponding author.

## Author Contributions

LW: data curation, investigation, formal analysis, writing—original draft, and writing—review and editing. SW: investigation and formal analysis. DH and LF: formal analysis, investigation, and methodology. YL and HL: project administration. WL: conceptualization, funding acquisition, project administration, supervision, conceptualization, data curation, funding acquisition, project administration, and supervision. All authors contributed to the article and approved the submitted version.

## Conflict of Interest

The authors declare that the research was conducted in the absence of any commercial or financial relationships that could be construed as a potential conflict of interest.

## Publisher’s Note

All claims expressed in this article are solely those of the authors and do not necessarily represent those of their affiliated organizations, or those of the publisher, the editors and the reviewers. Any product that may be evaluated in this article, or claim that may be made by its manufacturer, is not guaranteed or endorsed by the publisher.
