# Supplementary material for: Comprehensive Flavor Analysis of Volatile Components During the Vase Period of Cut Lily (Lilium spp. ‘Manissa’) Flowers by HS-SPME/GC–MS Combined With E-Nose Technology
Source: Front Plant Sci. 2022 Jun 17;13:822956. doi: 10.3389/fpls.2022.822956 (PMC9247614; doi:10.3389/fpls.2022.822956)
Supplement: Supplementary file 1 [file Table_1.DOCX]

**Table 1** Material type and performance description represented by sensors

| **Sensor number** | **Sensor name** | **Sensor sensitivity and general description** | **Detection limits**  **(mg· kg^−1^)** |
| --- | --- | --- | --- |
| 1 | W1C | Aromatic organic compounds | Toluene, 10 |
| 2 | W5S | Very sensitive, broad range sensitivity, reacts to nitrogen oxides | NO_2_, 1 |
| 3 | W3C | Ammonia, also used as sensor for aromatic compounds | Benzene, 10 |
| 4 | W6S | Detection on mainly hydrogen gas | H_2_, 0.1 |
| 5 | W5C | Alkanes, aromatic compounds | Propane, 1 |
| 6 | W1S | Sensitive to methane | CH4, 100 |
| 7 | W1W | Detection on inorganic sulfur compounds | H_2_S, 1 |
| 8 | W2S | Detection on alcohol | CO, 100 |
| 9 | W2W | Aromatic compounds, inorganic sulfur and organic compounds | H_2_S, 1 |
| 10 | W3S | Sensitive to methane and aliphatic organic compounds | CH_3_, 10 |
